# Design and analysis of a flexible Ruddlesden–Popper 2D perovskite metastructure based on symmetry-protected THz-bound states in the continuum

**DOI:** 10.1038/s41598-023-49224-9

**Published:** 2023-12-16

**Authors:** Seyedeh Bita Saadatmand, Samad Shokouhi, Vahid Ahmadi, Seyedeh Mehri Hamidi

**Affiliations:** 1 Faculty of Electrical and Computer Engineering, Tarbiat Modares University, Tehran, Iran; 2 Magneto-Plasmonic Lab, Laser and Plasma Research Institute, Shahid Beheshti University, Tehran, Iran

**Keywords:** Nanoscience and technology, Optics and photonics

## Abstract

A Ruddlesden–Popper 2D perovskite PEA_2_PbX_4_ (X = I, Br, and Cl) is proposed for metasurface applications. Density functional theory is used to analyze the optical, electrical, mechanical properties, moisture and thermodynamic stability of PEA_2_PbX_4_. The refractive index of PEA_2_PbX_4_ varies with the halides, resulting in 2.131, 1.901, and 1.842 for X = I, Br, and Cl, respectively. Mechanical properties with Voigt-Reuss-Hill approximations indicate that all three materials are flexible and ductile. Based on the calculations of formation energy and adsorption of water molecules, PEA_*2*_PbI_4_ has superior thermodynamic and moisture stability. We present a novel metasurface based on 2D-PEA_2_PbI_4_ and analyze symmetry protected-bound states in the continuum (sp-BIC) excitation. The proposed structure can excite multiple Fano quasi-BICs (q-BICs) with exceptionally high Q-factors. We verify the group theoretical analysis and explore the near-field distribution and far-field scattering of q-BICs. The findings indicate that x-polarized incident waves can excite magnetic toroidal dipole-electromagnetic-induced transparency-BIC and magnetic quadrupole-BIC, while y-polarized incident waves can excite electric toroidal dipole-BIC and electric quadrupole-BIC. The influence of meta-atom and substrate losses, array size limitations, and fabrication tolerances are also discussed. The proposed structure can be employed for applications in the THz region, such as polarization-dependent filters, bidirectional optical switches, and wearable photonic devices.

## Introduction

Terahertz (THz) technology is quite useful for screening security, and sensing without damaging or ionizing matter, as THz waves have properties such as low photon energy and strong penetrability^1^. Unfortunately, the weak interaction between light and matter in the THz region has hindered the development of THz technology due to the lack of powerful radiation sources^2,3^. However, by using resonant metasurfaces, it may be possible to overcome the challenges encountered in various THz research and improve the technology for diverse applications.

Metasurfaces possess resonances with a high-quality (Q) factor and concentrate light on a subwavelength scale, which makes them ideal for various applications, such as sensors, lasers, and nonlinear optics^4–7^. There have been numerous studies conducted on metasurface benefits in these fields. The conventional approach to achieving a high-Q resonance with an asymmetric spectral line shape is through effective design mechanisms that rely on Fano resonance. This type of resonance results from interference between a continuum state and a discrete state. A novel idea known as bound states in the continuum (BIC) has been put forth for structures lacking in-plane or out-of-plane inversion symmetry^8^. This concept highlights a correlation between the Q-factor of resonances and structural asymmetry, which can effectively be represented by an inverse-square law^9^. Symmetry-protected (sp) BIC arises due to the disallowed coupling between structures' eigenmodes and incident waves, owing to their mismatched symmetries. This leads to the formation of a localized state embedded within the continuum. The BIC ideally demonstrates an infinite Q value in a structure where symmetry is preserved. This renders it unobservable in the spectrum because of its zero spectral linewidth^10^. Asymmetry parameters can be used to engineer a quasi (q)-BIC mode with a finite linewidth and Q value. This approach offers a convenient means of accessing resonances with exceptionally high Q-factors^11^.

“The metamaterial absorbers (MMAs) are used to ensure high absorption of photons, which is required for absorbing a broad range of solar energy^12–14^. These MMAs are typically polarization-insensitive and maintain stable absorption levels even when subjected to mechanical stress or changes in incident angle. As a result, they can be widely utilized in a range of optical devices including sensors, solar cells, imaging tools, and detectors^15^.”

Metasurfaces composed of materials with high dielectric coefficients and low losses, are utilized to amplify the interaction between matter and light^16^. However, in the THz range, only a limited number of materials possess such properties. Among the dielectric metasurfaces described in the literature, silicon^17,18^ and lithium tantalate^19,20^ are frequently employed due to their desirable characteristics. Although there have been advancements in the THz region, it is still critical to introduce and examine novel materials. Furthermore, flexible metastructures are constructed by depositing metals on flexible substrates^21^. This approach results in significant radiation losses and a decrease in the Q-factor. Consequently, exploring the potential of employing new and flexible dielectric materials as an alternative to metals in the metastructure becomes crucial.

Ruddlesden–Popper quasi-two-dimensional (2D) perovskites can be described by the chemical formula (L)_2_(A)_n−1_BX_3n+1_, where L is a large monovalent cation (i.e., aliphatic or aromatic alkylammonium), A represents a small cation (such as formamidinium, methylammonium, or cesium), B indicates a divalent cation (i.e., tin and lead), X represents a halide anion (such as iodine (I), bromine (Br) and chlorine (Cl)), and n is the number of lead halide octahedral layers. For n = 1, the perovskite structure is pure 2D with formula L_2_BX_4_, and for n = ∞, it becomes analogous to bulk or three-dimensional (3D) perovskite with formula ABX_3_^22^. Recently, 3D perovskites have been utilized in sensing applications^23–25^.

The 2D perovskites exhibit unique and remarkable structural and physical properties, such as low cost, ease of fabrication, direct and tunable bandgap, a soft and dynamic structure, and a relatively high nonlinear refractive index. Unlike 3D perovskites, the flexibility of the organic layer in 2D perovskites further contributes to their softness, along with the Van der Waals interface. In addition, 2D perovskite materials exhibit higher environmental and chemical stability compared to their 3D counterparts^26^. In the 2D perovskite material PEA_2_PbX_4_ (X = I, Br, and Cl), Phenethylammonium (PEA) with the chemical formula C_6_H_5_(CH_2_)_2_NH_3_ is used as the large cation. These aromatic cations exhibit hydrophobic characteristics typical of bulky aromatic cations. Consequently, they not only improve the material's environmental stability but also can influence its mechanical properties. In layered 2D halide perovskites based on PEA, moisture stability is generated by the hydrophobic nature of aromatic organic ammonium spacer cations. In general, perovskite materials, especially 2D perovskite (i.e., PEA_2_PbX_4_), have more flexibility than silicon because they have smaller elastic moduli^27,28^. PEA_2_PbX_4_ has recently been used in many optoelectronic fields due to its outstanding properties, such as moisture stability, photostability, and π-π interaction^29^, and we want to introduce and study it in the THz region for the first time to the best of our knowledge.

In the present work, we employ first-principles density functional theory (DFT) analysis to examine the mechanical, optical, electrical, and stability characteristics of PEA_2_PbX_4_ (X = I, Br, and Cl) to investigate their potential use in THz applications. The results indicate that PEA_2_PbI_4_ is significantly more thermodynamically stable than PEA_2_PbBr_4_ and PEA_2_PbCl_4_, and is some orders of magnitude more stable than 3D perovskites. The DFT calculation is used to assess the shear modulus, Poisson's ratio, Young's modulus, and bulk modulus of these compounds. The elastic moduli calculated by the Voigt-Reuss-Hill approximations indicate that these compounds have both ductility and mechanical stability. Moreover, for the first time, we demonstrate the moisture stability of PEA_2_PbX_4_ by calculating the adsorption energy of water molecules. We find that these materials have a high dielectric constant, near-zero loss in the THz region, thermodynamic stability, moisture resistance, and flexibility.

Following that, we introduce a novel kind of metastructure that uses periodic circular slot rings in a Ruddlesden–Popper quasi-2D perovskite layer (PEA_2_PbI_4_), which has not been reported yet to the best of our knowledge. By introducing an offset distance of the inner ring from the center, the symmetry of the structure is disrupted, which opens a zero-order channel, enabling the conversion of the dark modes to q-BIC states with a finite linewidth. After conducting an extensive mathematical examination utilizing group theory and finite element eigenfrequency calculations, we prove that the proposed metastructure can excite multiband high-quality q-BICs, such as the magnetic toroidal dipole (MTD) and electric toroidal dipole (ETD), which exhibit specific symmetry properties. By calculating the far-field radiations from the multipole decomposition method and near-field analysis with finite element frequency domain calculations, a thorough study is conducted on the q-BIC characteristics. The analysis of symmetry is beneficial in determining which BICs can be excited based on the polarization of the incident wave. Moreover, when the broad mode is coupled to the q-BIC, the Fano peak of low bandwidth transparency is visible. The Q value of electromagnetic-induced transparency-BIC resonance can be regulated by adjusting the offset distance. This work presents a valuable reference for developing applications in the THz region such as polarization-dependent switches, multi-channel wearable biochemical sensing, notch polarization-dependent filters, and non-linear optics.

## Materials and methods

**Figure 1:**
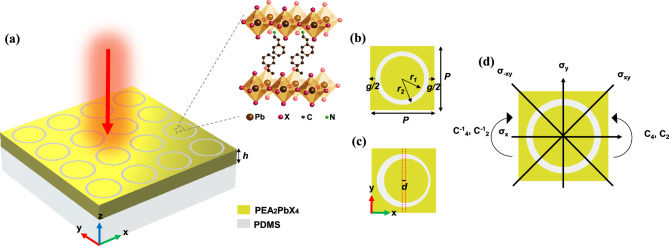
Schematic of (**a**) the proposed structure, (**b**) symmetric UC, (**c**) asymmetric UC, and (**d**) bases of UC’s symmetry.

The schematic crystal structure of PEA_2_PbX_4_ (X = I, Br, and Cl) is depicted in Fig. 1a. Table S1 presents crystal data for room temperature structures.

### Optical properties

The first-principle analysis based on DFT is used to compute the complex refractive index (RI) of PEA_2_PbX_4_ (X = I, Br, and Cl). The dielectric function, denoted as ε(ω) = ε_1_(ω) + iε_2_(ω), can be expressed as a complex quantity with a real part ε_1_(ω) and an imaginary part ε_2_(ω). To obtain optical properties, the imaginary part of the dielectric function is computed using the momentum matrix elements that correspond to the unoccupied and occupied wave functions in agreement with the selection rules, and is expressed as^30^:

1 $$ \varepsilon_{2} \left( {\upomega } \right) = \frac{{2e^{2} \pi }}{{{\Omega }\varepsilon_{0} }}\mathop \sum \limits_{k,v,c} \left| { < \psi_{k}^{c} } \right|u.r\left| {\psi_{k}^{v} > } \right|^{2} \delta \left( {E_{k}^{c} - E_{k}^{v} - \hbar {\upomega }} \right) $$

where *ħω* is the photon energy, *e* represents the electronic charge, *ε*_*0*_ is the dielectric constant in vacuum,* Ω* is the volume of a unit cell, *u* is the unit vector along the polarization of the incident electric field, and *ψ*_*k*_^*v*^ and *ψ*_*k*_^*c*^ are wave functions for valence and conduction band electrons at a certain wave number, respectively. By employing the Kramers–Kronig relations, it is possible to extract the real component of the dielectric function from the imaginary component. The relation between complex dielectric function and complex RI is^30^:

2 $$ \varepsilon_{1} \left( {\upomega } \right) = n^{2} - k^{2} $$

3 $$ \varepsilon_{2} \left( {\upomega } \right) = 2nk $$

where *k* and *n* are the imaginary and real parts of the refractive index, respectively (see Sect. S1).

### Thermodynamic stability

The formation energy (*FE*) of quasi-2D perovskite ($L_{2} A_{n - 1} B_{n} X_{{3{\text{n}} + 1}}$) is defined as^31^:

4 $$ FE\left( {L_{2} A_{n - 1} B_{n} X_{{3{\text{n}} + 1}} } \right) = E\left( {L_{2} A_{n - 1} B_{n} X_{{3{\text{n}} + 1}} } \right) - 2E\left( {LX} \right) - \left( {{\text{n}} - 1} \right){\text{E}}\left( {{\text{AX}}} \right) - {\text{nE}}\left( {{\text{B}}X_{2} } \right) $$

where *E* is the total energy of the component material. For pure 2D perovskites (n = 1), the equation is modified as:

5 $$ FE\left( {L_{2} BX_{4} } \right) = E\left( {L_{2} BX_{4} } \right) - 2E\left( {LX} \right) - {\text{E}}\left( {{\text{B}}X_{2} } \right) $$

More negative formation energies directly correlate with a higher level of thermodynamic stability in the system^31^.

### Mechanical properties

The mechanical properties of perovskites are critical for the analysis of ductility and flexibility in various applications. The mechanical properties are calculated using the stress–strain method to compute the elastic constant elements (*C*_*ij*_). From Hooke's law, the stress (*σ*_*i*_) and strain (*ϵ*_*j*_) in Voigt notation are related as^32^:

6 $$  \sigma_{j} = C_{ij} \epsilon_{j} $$

The shear modulus (*G*), bulk modulus (*B*), Young’s modulus (*E*), and Poisson’s ratio (*τ*) are calculated by DFT analysis. Employing Voigt (*B*_*V*_*, G*_*V*_*, E*_*V*_*, ν*_*V*_) and Reuss (*B*_*R*_*, G*_*R*_*, E*_*R*_*, ν*_*R*_) approximations, we can define the relations of elastic moduli by^33–36^:

7 $$ B_{V} = \frac{1}{9}\left[ {\left( {C_{11} + C_{22} + C_{33} } \right) + 2\left( {C_{12} + C_{13} + C_{23} } \right)} \right] $$

8 $$ G_{V} = \frac{1}{15}\left[ {\left( {C_{11} + C_{22} + C_{33} } \right) - \left( {C_{12} + C_{13} + C_{23} } \right) + 3\left( {C_{44} + C_{55} + C_{66} } \right)} \right] $$

9 $$ E_{V} = \frac{{9B_{V} G_{V} }}{{3B_{V} + G_{V} }} $$

10 $$ \nu_{V} = \frac{{3B_{V} - 2G_{V} }}{{2\left( {3B_{V} + G_{V} } \right)}} $$

11 $$ B_{R} = \frac{1}{{\left( {S_{11} + S_{22} + S_{33} } \right) + 2\left( {S_{12} + S_{13} + S_{23} } \right)}} $$

12 $$ G_{R} = \frac{15}{{[4\left( {S_{11} + S_{22} + S_{33} } \right) + 3\left( {S_{44} + S_{55} + S_{66} } \right) - 4\left( {S_{12} + S_{13} + S_{23} } \right)}} $$

13 $$ E_{R} = \frac{{9B_{R} G_{R} }}{{3B_{R} + G_{R} }} $$

14 $$ \nu_{R} = \frac{{3B_{R} - 2G_{R} }}{{2\left( {3B_{R} + G_{R} } \right)}} $$

The inverse of the elastic constant (*C*_*ij*_) is denoted as *S*_*ij*_. The Hill approximation *(B*_*H*_*, G*_*H*_*, E*_*H*_*, ν*_*H*_*)* is the average of the Voigt and Reuss methods^36^:

15 $$ B_{H} = \frac{{B_{V} + B_{R} }}{2}, G_{H} = \frac{{G_{V} + G_{R} }}{2},E_{H} = \frac{{E_{V} + E_{R} }}{2},\nu_{H} = \frac{{\nu_{V} + \nu_{R} }}{2} $$

Pugh's ratio refers to the ratio of the bulk modulus to the shear modulus (B/G). If the ratio of B/G is above 1.75, the material is characterized as ductile; otherwise, it is considered brittle. Another parameter for separating ductile from brittle materials is Poisson’s ratio. Ductility is observed in materials with a Poisson's ratio greater than 0.26, whereas materials with a Poisson's ratio of 0.26 or lower exhibit brittleness. Generally, materials with a higher Poisson’s ratio are more ductile^37^.

### Moisture stability

The adsorption energy of water on perovskites is calculated by^38^:

16 $$ E_{ads} = E_{adsorbate/sub} - \left[ {E_{adsorbate} + E_{sub} } \right] $$

where *E*_*adsorbate*_ presents the total energy of adsorbate (i.e., water molecules), *E*_*sub*_ is the total energy of the isolated substrate system (i.e., perovskite surface), and *E*_*adsorbate/sub*_ is the total energy of adsorption system (i.e., water on the perovskite surface). For the negative value of *E*_*ads*_, perovskite has hydrophilicity. On the other hand, for a positive *E*_*ads*_ value, perovskite has hydrophobicity.

### Structure design and symmetry analysis

The proposed metastructure comprises a PDMS substrate (n = 1.4) with a thin layer of Ruddlesden–Popper perovskite on top, which has a periodic arrangement of slot rings (Fig. 1a). The symmetric and asymmetric unit cells (UC) are illustrated in Fig. 1b and c, respectively, and are characterized by certain structural features such as the inner and outer radii of the rings (*r*_*1*_ = 67.5 µm and *r*_*2*_ = 75 µm), the gap between adjacent rings (*g* = 31.25 µm), the thickness of the perovskite layer (*h* = 72.5 µm), and the period of the UC (*P* = 181.25 µm). By adjusting the inner ring position, an asymmetric structure is achieved. The offset distance of the inner ring from the center is denoted by *d*. Typically, the lithography process affects mainly the roughness and uniformity of the structure while the position of the rings remains fixed. This allows precise control over the asymmetry parameter during experimentation. This approach does not change the volume of the material portion of the metasurface, resulting in a minor shift in resonance frequency that is mainly caused by variations in the coupling between neighboring rings. Overall, this method results in a relatively stable resonance frequency for q-BICs. To determine the optical characteristics of the metastructure, the finite element method is utilized. Floquet-Bloch periodic boundary conditions are used along the x–z and y–z planes. Additionally, two perfectly matched layers are positioned at a specified distance from the structure along the z-axis and are supported by scattering boundary conditions.

The unperturbed structure's UC is depicted in Fig. 1d, displaying a 2D group of geometrical symmetry denoted as C_4v_ in Schoenfies notation^39^. Table 1 shows the four dark modes with irreducible representations (IRREPs) A_1_, A_2_, B_1_, and B_2_, and two degenerate bright modes (E), which are supported by the C_4v_ symmetric group. Table 2 displays the resonant frequencies and field profiles associated with each mode. It should be noted that we use PEA_2_PbI_4_ in eigenfrequency calculations; the reason for this will be explained in the next sections. Four dark modes (i.e., real eigenfrequency) have different symmetries compared to the incident electric field and cannot be excited by it, as they belong to different IRREPs^40^. Because their components are orthogonal to that of the electric field and cannot be activated by incident waves. The arrow plots presented in Table 2 provide a visual representation indicating that these dark modes lack a total electric dipole moment in the x–y plane, thereby making them incapable of being coupled with the excited plane wave. Additionally, IRREPs E (E_11_, E_12_) describe two more orthogonal modes (D_y_ and Q_x_), while the other elements (E_21_, E_22_) specify two more orthogonal modes (D_x_ and Q_y_). When subjected to a 90-degree rotation, D_x_ and Q_y_ transform into D_y_ and Q_x_, respectively. This establishes the polarization-independence of E modes.

**Table 1:** IRREPs of group C_4v_.

C_4v_	e	C_2_	C_4_	C^-1^_4_	σ_x_	σ_y_	σ_xy_	σ_-xy_	Mode type
A_1_	1	1	1	1	1	1	1	1	Radial
A_2_	1	1	1	1	− 1	− 1	− 1	− 1	Circular
B_1_	1	1	− 1	− 1	1	1	− 1	− 1	Quadrupole
B_2_	1	1	− 1	− 1	− 1	− 1	1	1	Quadrupole
E	2	− 2	0	0	0	0	0	0	Dipole/quadrupole

**Table 2:** Eigenfrequencies of the proposed structure with C_4v_ symmetry.

IRREP	Mode schematic	Electric field profile	Magnetic field profile	Mode type
**A**_**1**_ f_res_ = 0.99240 THz				Radial mode
**A**_**2**_ f_res_ = 0.88074 THz				Circular mode
**B**_**1**_ f_res_ = 1.00258 THz				Quadrupole mode
**B**_**2**_ f_res_ = 0.98245 THz				Quadrupole mode
**E**_**11**_**, E**_**12**_ f_res1_ = 0.993 + 0.0004i THz f_res2_ = 0.8999 + 0.0006i THz				Dipole and quadrupole mode (D_x_ and Q_y_)
		
**E**_**21**_**, E**_**22**_ f_res1_ = 0.993 + 0.0004i THz f_res2_ = 0.8999 + 0.0006i THz				Dipole and quadrupole mode (D_y_ and Q_x_)
		

To excite the dark resonances, it is necessary to reduce the symmetry of the UC. However, reducing the symmetry from C_4v_ to C_2v_ will not excite the dark modes. This conclusion is supported by Table S3 and Fig. S3 which display the IRREPs and electric field profiles of the C_2v_ group, and Table S4, which outlines the process of symmetry degeneration from C_4v_ to lower groups. However, it is possible to excite the dark resonances by reducing the symmetry of C_4v_ to C_s_. The IRREPs of C_s_ are presented in Table 3. This reduction allows the existence of corresponding electric field components. In this case, according to Table S4, A_1_ and B_1_ (A_2_ and B_2_) of C_4v_ are reduced to A (B) of C_s_.

**Table 3:** IRREPs of group C_s_.

C_s_	e	σ_x_
A	1	1
B	1	− 1

The symmetries of distinct non-degenerate modes correspond to different incident wave polarizations, resulting in polarization-dependent BIC modes even for the same perturbation type. As explained in Sect. S4 and shown in Fig. S4, BIC modes A_1_ and B_1_ of C_4v_ can be excited by an x-polarized plane wave, whereas BIC modes A_2_ and B_2_ can be excited by y-polarization, which indicates the possibility of selectively exciting BIC modes. Therefore, BICs are polarization-dependent.

## Results and discussions

### Material property studies

**Figure 2:**
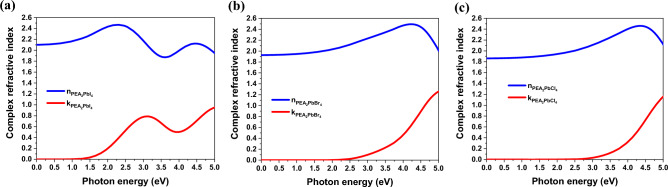
Complex RI curves vs. frequency for (**a**) PEA_2_PbI_4_, (b) PEA_2_PbBr_4_, and (**c**) PEA_2_PbCl_4_.

Complex RIs of PEA_2_PbX_4_ are shown in Fig. 2a–c. These materials exhibit neither dispersion nor loss in the THz region, making them suitable for employment in this spectral range. It is worth mentioning that the RI decreases as the halide changes from iodine to bromine and then to chlorine. This is because of the increase in the bandgaps (see Fig. S5).

Table 4 presents the mechanical properties of PEA_2_PbX_4_ using the Voigt-Reuss-Hill approximations. The table shows that all three structures are mechanically stable (see Sect. S2) and ductile with B/G > 1.75 or *ν* > 0.26 according to DFT calculations. The bulk, shear, and Young’s moduli exhibit an increase when the halogen atom changes from I to Br and then to Cl. This indicates that PEA_2_PbI_4_ is more flexible compared to PEA_2_PbBr_4_ and PEA_2_PbCl_4_. Notably, silicon and GaAs have very high Young's modulus values of 174.8 GPa and 87 GPa, respectively, making them stiffer than PEA_2_PbX_4_, which exhibits Young's modulus range of 12–22 GPa^41^. Therefore, PEA_2_PbX_4_-based metasurfaces can be used in wearable photonic devices. In addition, the relatively low bulk modulus of all three structures suggests their inherent softness, enabling easy transformation into thin films. This characteristic is particularly crucial for photonic applications.

**Table 4:** Mechanical properties of PEA_2_PbX_4_.

Approach		PEA_2_PbI_4_	PEA_2_PbBr_4_	PEA_2_PbCl_4_
Voigt	${\text{B}}_{{\text{V}}} {\text{(GPa)}}$	12.024	13.625	14.173
	${\text{G}}_{{\text{V}}} {\text{(GPa)}}$	7.050	7.058	7.125
	${\text{E}}_{{\text{V}}} {\text{(GPa)}}$	17.692	18.056	18.307
	${\text{B}}_{{\text{V}}} {\text{/G}}_{{\text{V}}}$	1.705	1.930	1.989
	${\upnu }_{{\text{V}}}$	0.254	0.279	0.284
	Type of material	Brittle	Ductile	Ductile
Reuss	${\text{B}}_{{\text{R}}} {\text{(GPa)}}$	11.354	13.459	13.942
	${\text{G}}_{{\text{R}}} {\text{(GPa)}}$	5.473	6.822	6.755
	${\text{E}}_{{\text{R}}} {\text{(GPa)}}$	14.146	17.509	17.447
	${\text{B}}_{{\text{R}}} {\text{/G}}_{{\text{R}}}$	2.074	1.972	2.063
	${\upnu }_{{\text{R}}}$	0.292	0.283	0.291
	Type of material	Ductile	Ductile	Ductile
Hill	${\text{B}}_{{\text{H}}} {\text{(GPa)}}$	11.689	13.542	14.058
	${\text{G}}_{{\text{H}}} {\text{(GPa)}}$	6.261	6.940	6.940
	${\text{E}}_{{\text{H}}} {\text{(GPa)}}$	15.939	17.782	17.878
	${\text{B}}_{{\text{H}}} {\text{/G}}_{{\text{H}}}$	1.866	1.951	2.024
	${\upnu }_{{\text{H}}}$	0.272	0.281	0.288
	Type of material	Ductile	Ductile	Ductile

Table 5 investigates the thermodynamic stability of PEA_2_PbX_4_ by calculation of the formation enthalpy energy for its component materials. As can be seen, PEA_2_PbI_4_ has a more negative value of formation enthalpy energy. This material is about 2 times more stable than PEA_2_PbBr_4_ and PEA_2_PbCl_4_, respectively, and is some orders of magnitude more stable than 3D perovskites^42^.

**Table 5:** Formation enthalpy energies of PEA_2_PbX_4_.

2D perovskite	E(PEA_2_PbX_4_) (eV)	2*E(PEAX) (eV)	E(PbX_2_) (eV)	FE (eV)
PEA_2_PbI_4_	− 12,655.514	− 8076.481	− 4574.344	− 4.689
PEA_2_PbBr_4_	− 26,157.016	− 16,577.434	− 9575.247	− 4.335
PEA_2_PbCl_4_	− 26,839.994	− 16,919.679	− 9915.109	− 4.506

Table 6 shows the adsorption energy of water molecules on PEA_2_PbX_4_ (see Sect. S3). The proposed materials exhibit better hydrophobicity than most perovskites, making them more moisture-stable^43^. PEA_2_PbI_4_ adsorbs water molecules approximately four times less than conventional 3D perovskites^44^. Among the proposed materials, PEA_2_PbI_4_ exhibits the highest stability, whereas PEA_2_PbBr_4_ shows the lowest stability against moisture. Therefore, adsorption energy and formation energy are directly correlated, and materials that have higher thermodynamic stability also tend to have better moisture stability^45^.

**Table 6:** Adsorption energy for PEA_2_PbX_4_.

2D perovskite	E_water/perovskite_ (eV)	E_perovskite_ (eV)	E_water_ (eV)	E_ads_ (eV)
PEA_2_PbI_4_	− 13,123.759	− 12,654.890	− 468.804	− 0.065
PEA_2_PbBr_4_	− 26,624.216	− 26,155.335		− 0.077
PEA_2_PbCl_4_	− 27,307.211	− 26,838.479		− 0.072

Based on the above results, we chose PEA_2_PbI_4_ for the metastructure due to its higher RI in the THz region, superior thermodynamic stability compared to PEA_2_PbBr_4_ and PEA_2_PbCl_4_, better moisture stability, and ductile nature.

### Wave propagation studies and mode analysis

**Figure 3:**
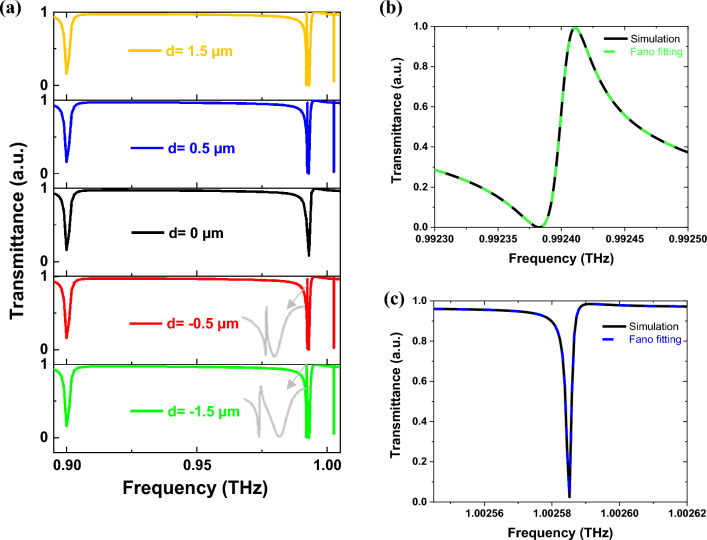
(**a**) The transmittance spectrum under x-polarization with various *d*, (**b**) Fano fitting of A_1_ at *d* = 0.5, and (**c**) Fano fitting of B_1_ at *d* = 0.5.

**Figure 4:**
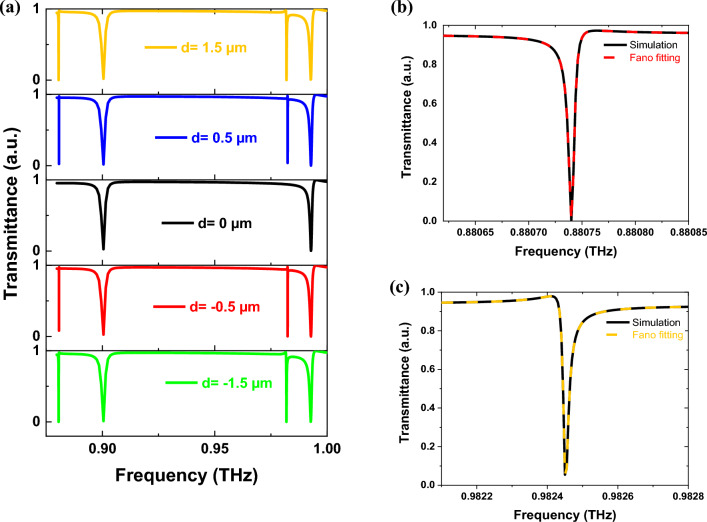
(**a**) The transmittance spectrum under y-polarization with various *d*, (**b**) Fano fitting of A_2_ at *d* = 0.5, and (c) Fano fitting of B_2_ at *d* = 0.5.

If *d* = 0, the structure exhibits C_4v_ symmetry, and its transmittance spectrum for x and y polarizations is depicted in Figs. 3a and 4a, respectively. For both polarizations, two bright modes belonging to IRREP E are observed. Since q-BICs do not exist at *d* = 0, there is no energy leakage from the bound states to the zero-order channel. Furthermore, these modes are positioned at identical frequencies under both x and y polarizations, with their fields rotating 90 degrees from each other, highlighting their degeneracy (see Fig. S6). By adding an offset, it becomes possible to couple x-polarized waves with A_1_ and B_1_, and y-polarized waves with A_2_ and B_2_. This has been identified in the group theory analysis in “Structure design and symmetry analysis” section. As a consequence, the excitation of multiband q-BICs is selectively achieved depending on the polarization of the incident waves. In addition, symmetry breaking leads to the emergence of a transmission peak (mode A_1_) within the near-zero transmittance valley of the non-BIC mode under x-polarization. The resonant frequency of A_1_ is almost identical to that of the bright mode, producing an electromagnetic-induced transparency (EIT) effect. Moreover, the resonance peaks of q-BIC become broader as *d* moves away from zero. The typical Fano formula is utilized for fitting the Fano resonance curves of q-BICs^46^:

17 $$ T_{{{\text{Fano}}}} (\omega ) = \left| {a_{1} + ja_{2} + \frac{b}{{\omega - \omega_{0} + j\gamma }}} \right|^{2} $$

where *ω*_*0*_ is the resonant frequency, *a*_*1*_, *a*_*2*_, and *b* are the constants, and *γ* is the total rate of damping that characterizes the Q-factor (${\text{Q} = \omega }_{{0}} {{/2\gamma }}$) of the q-BICs. Figures 3b, c and 4b, c illustrate the fitting results for the four q-BICs at *d* = 0.5 µm. The Q-factors at *d* = 0.5 µm are 1.2 ${{ \times }}$ 10^4^, 5 ${{ \times }}$ 10^5^, 4.7 ${{ \times }}$ 10^5^, and 4 ${{ \times }}$ 10^4^ for A_1_, B_1_, A_2_, and B_2_, respectively.

The absence of clutter modes in the transmittance spectrum prevents interference with resonant states in the desired frequency range while maintaining high Q-factor q-BIC modes. Furthermore, all modes in this frequency range exhibit a spectral contrast ratio and modulation depth of approximately 100%. The spectral contrast ratio is defined as [(T_on_ − T_off_)]/[(T_on_ + T_off_)] × 100% and modulation depth is defined as [(T_on_ − T_off_)/T_on_] × 100%, where T_off_ and T_on_ represent the minimum and maximum transmittance, respectively^47^. These remarkable modulation depths and spectral contrast ratios significantly improve detection accuracy and switching efficiency in sensor and switch applications.

**Figure 5:**
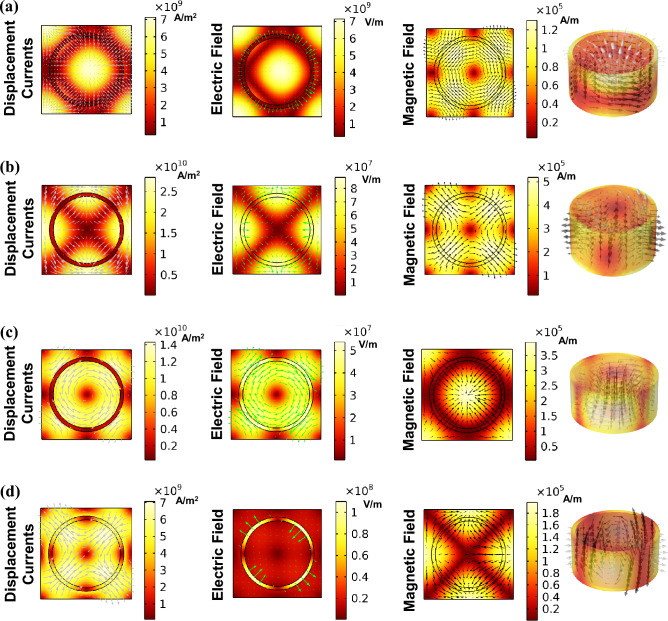
The color maps of displacement currents, electric, and magnetic fields, as well as vector distributions in the x–y plane for (**a**) A_1_, (**b**) B_1_, (**c**) A_2_, and (**d**) B_2_. The fourth column displays a 3D representation of the electric field.

To provide an intuitive representation of the resonances, we analyze the near-field distributions of the displacement current, electric, and magnetic field within the UC shown in Fig. 5. In mode A_1_, the head-to-tail configuration of magnetic moments is quite evident. The presence of current loops in the plane perpendicular to it provides clear indications of the magnetic toroidal dipole (MTD) mode (also see Fig. S7a). This mode can be considered as the combined result of both intra-UC and inter-UC moments. Non-parallel magnetic moments are evident in mode B_1_, which represents a magnetic quadrupole (MQ). In the case of A_2_, the field map reveals a clear vortex of displacement currents threading through the inner ring. Additionally, magnetic moments take the form of a vortex within the plane perpendicular to the current. This configuration enables easy recognition of an electric toroidal dipole (ETD) (also see Fig. S7b). Mode A_2_ can also be considered as the combined result of both intra-UC and inter-UC moments. Finally, in B_2_, an electric quadrupole (EQ) mode can be identified by antiparallel electric moments. The fourth column in Fig. 5 displays a 3D representation of the electric field. The displacement current is represented by gray arrows, while the magnetic field is depicted by black arrows.

**Figure 6:**
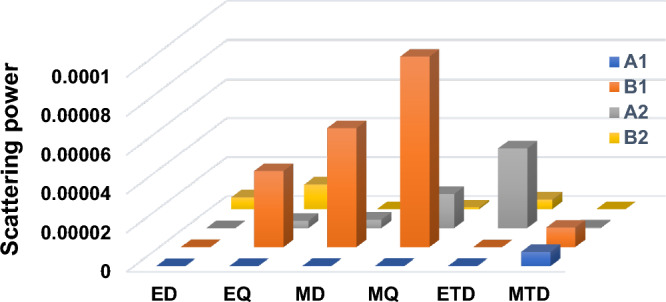
The contributions of q-BICs to scattering power.

To perform a more comprehensive investigation of q-BIC properties, we utilize the Cartesian multipole decomposition technique (see Sect. S7)^48^. During this process, we integrate the displacement current density within the UC, to gain insight into the distribution of the electromagnetic source in the far-field. According to Fig. 6, the dominant multipole components in A_1_, B_1_, A_2_, and B_2_ are MTD, MQ, ETD, and EQ, respectively. These results are completely consistent with Fig. 5.

**Figure 7:**
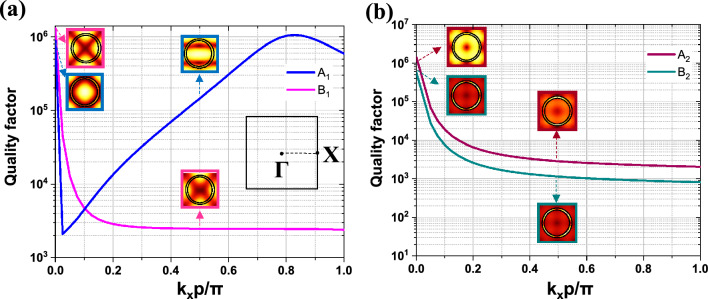
The Q values of (**a**) A_1_ and B_1_, and (**b**) A_2_ and B_2_ along the path from Γ to X in the first Brillouin zone.

The verification of the four q-BIC modes' symmetry-protected nature is supported by an eigenvalue analysis of a part of their band diagram. In this analysis, the k_x_ component of the periodic boundary condition sweeps from 0 to π/p (Γ to X in the first Brillouin zone). As shown in Fig. 7a and b, while all four modes theoretically have ultra-high Q-factors at the Γ point in the first Brillouin zone, their values decrease sharply from the Γ point, demonstrating their symmetry protected-bound state in the continuum (sp-BIC) nature. Moreover, in Fig. 7a, we observe a high Q-factor at k_x_ = 0.8π/p, which corresponds to off Γ-BIC and lies beyond the scope of this work. The electric field profile of the modes at k_x_ = 0 and k_x_ = 0.5π/p can be observed in the insets of Fig. 7a and b. It is evident that the nature of each mode remains unchanged despite varying k_x_, however, the Q-factors differ.

**Figure 8:**
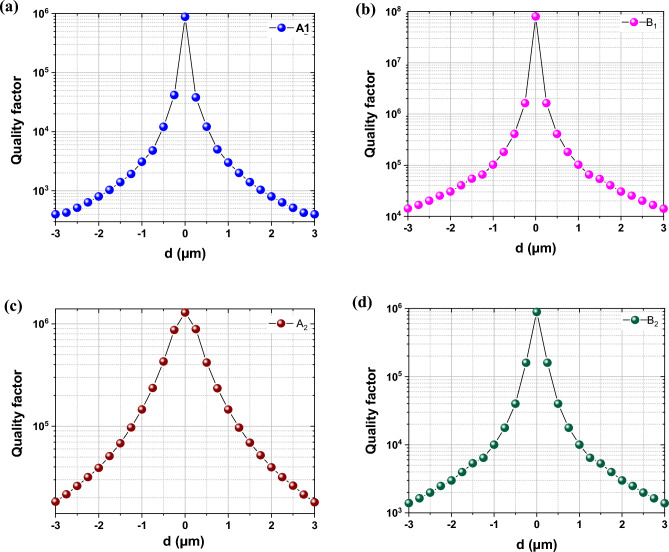
Q values of (**a**) A_1_, (**b**) B_1_, (**c**) A_2_, and (**d**) B_2_ as a function of the inner ring’s offset distance from the center.

We now examine how the Q value of the q-BICs is influenced by the offset distance of the inner ring from the center, as illustrated in Fig. 8. The results reveal that the Q-factor of the q-BICs exhibit remarkably high sensitivity to changes in *d*. When *d* deviates from 0, we observe an exponential decrease in the Q-factor (Q ∝ *d*^−2^). For instance, at *d* = 0.5 µm, the Q-factor is significantly higher compared to that of *d* = 1 or − 1 µm, with a difference of nearly four orders of magnitude. This exponential decline in the Q-factor of the q-BICs agrees well with findings from previous studies where an increase in the asymmetry parameter resulted in a similar trend^49,50^. Consequently, manipulation of the Q value of q-BIC modes can be realized with asymmetric parameters.

### Considerations of fabrication non-idealities

**Figure 9:**
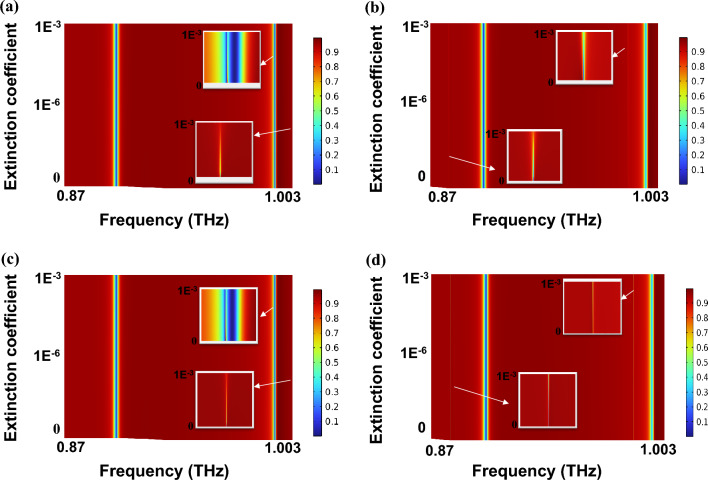
Evolution of the transmittance spectra versus meta-atom’s extinction coefficient for (**a**) x-polarized incident wave, (**b**) y-polarized incident wave at *d* = 0.5 µm, the evolution of the transmittance curves versus substrate’s extinction coefficient for (c) x-polarized incident wave, and (d) y-polarized incident wave at *d* = 0.5 µm.

PEA_2_PbI_4_ has extremely low absorption losses in the THz region. However, in the real fabrication process, surface roughness and defects can lead to absorption and scattering losses, which are major concerns when designing the metasurface. These losses are considered by adding the imaginary part of the PEA_2_PbI_4_ refractive index (*k*) (i.e., the extinction coefficient) as *n*_*PEA2PbI4*_ = *n-jk.* Figure 9a and b and their insets show that for *d* = 0.5 µm as the *k* of PEA_2_PbI_4_ rises, particularly when *k* approaches 10^–3^, there is a noticeable increase in the full width at half maximum (FWHM) of q-BICs and a reduction in transmittance intensity. However, the transmittance spectrum remains largely unaffected when k $\le$ 10^−8^. Meanwhile, the FWHM of non-BICs does not change, and all resonance frequencies exhibit no shifts. The impact of meta-atom losses is more pronounced on reflectance curves compared to transmittance curves (see Fig. S8). The results in Fig. 9c and d show that substrate losses have a notably smaller impact than meta-atom losses. Consequently, the observed variations in Q-factor and transmittance intensity of q-BICs are relatively minor.

**Figure 10:**
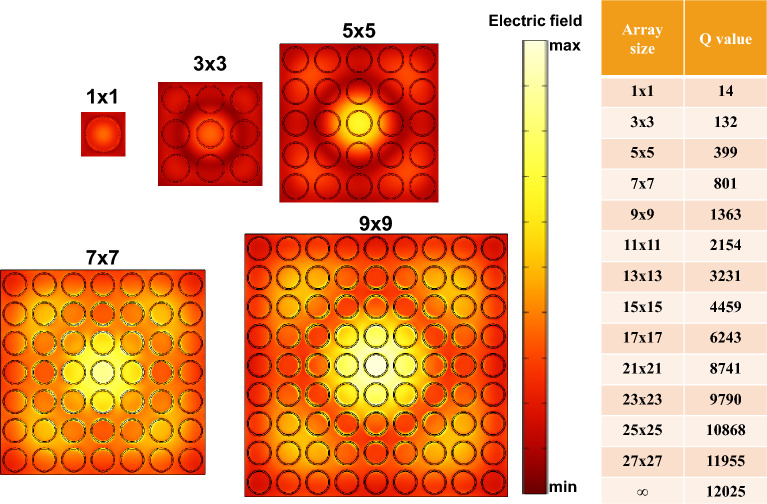
Electric Field Profiles of A_1_ in finite-sized arrays: 1 × 1, 3 × 3, 5 × 5, 7 × 7, and 9 × 9. The table displays the A_1_ Q value in different array sizes.

In the experiment, the actual Q-factor can be limited by the finite size of the array^51^. Periodicity among meta-atoms suppresses radiative loss through near-field coupling. To investigate the near-field coupling among the meta-atoms, we simulate how the resonance evolves for different array sizes (the number of UCs in the metastructure). The results for A_1_ are presented in Fig. 10. The electric field magnitude rises in proportion to the size of the array. Moreover, the central rings of the array demonstrate a greater level of field compared to those located at the edges. When a 27 × 27 array size is employed, the Q-factor of metastructure is very close to that of an infinite array. To achieve a Q value comparable to that of an infinite structure, the minimum array sizes for modes B_1_, A_2_, and B_2_ are 15 × 15, 17 × 17, and 25 × 25, respectively (not shown here).

To examine the impact of fabrication process tolerance on the q*-*BICs of the proposed structure, we conducted investigations into the Q*-*factor and resonance frequency of the modes within a ${{ \pm }}$ 5% range of change in the structure parameters. Based on the findings presented in Table 7, it can be observed that when the parameters of the metastructure undergo a ${{ \pm }}$ 5% change, the Q-factors of q-BICs remain in the same order. Consequently, in practical scenarios, one can expect to observe high Q-factors. To discuss the red and blue shift of the modes we can refer to the dielectric resonator's Mie resonance frequency explained as^52^:

18 $$ f = \frac{{\theta c^{2} }}{{2\pi V(x,y,z)\sqrt {\mu \varepsilon } }} $$

where V (x, y, z) is related to the size of the resonator, while μ and ε represent its permeability and permittivity, respectively. In addition, θ is a constant value that applies to a specific resonance. The rise (fall) in height, radius, and period can cause the device volume V (x, y, z) to increase (decrease), and as a result, the resonant frequency redshifts (blueshifts). Therefore, the shift in resonance frequencies caused by the aforementioned parameters is consistent with the Mie theory.

**Table 7:** The effect of fabrication tolerance on resonance frequencies and Q-factor.

Fabrication tolerance		f_res_ (P/h/r) THz	f_res_ (P/h/r-5%) THz	f_res_ (P/h/r + 5%) THz	Q-factor (P/h/r)	Q-factor (P/h/r − 5%)	Q-factor (P/h/r + 5%)
P ${{ \pm }}$ 5%	A_1_	0.99240	1.0267	0.95879	1.2 ${{ \times }}$ 10^4^	1.1 ${{ \times }}$ 10^4^	2.2 ${{ \times }}$ 10^4^
	B_1_	1.00258	1.04608	0.96358	4.7 ${{ \times }}$ 10^5^	4.6 ${{ \times }}$ 10^5^	4.8 ${{ \times }}$ 10^5^
	A_2_	0.88074	0.92089	0.84353	5 ${{ \times }}$ 10^5^	3.8 ${{ \times }}$ 10^5^	5.6 ${{ \times }}$ 10^5^
	B_2_	0.98245	1.02438	0.93312	4 ${{ \times }}$ 10^4^	2.7 ${{ \times }}$ 10^4^	5 ${{ \times }}$ 10^4^
h ${{ \pm }}$ 5%	A_1_	0.99240	1.00049	0.97023	1.2 ${{ \times }}$ 10^4^	1 ${{ \times }}$ 10^4^	4 ${{ \times }}$ 10^4^
	B_1_	1.00258	1.01158	0.98600	4.7 ${{ \times }}$ 10^5^	4.5 ${{ \times }}$ 10^5^	5.2 ${{ \times }}$ 10^5^
	A_2_	0.88074	0.88404	0.87188	5 ${{ \times }}$ 10^5^	3.8 ${{ \times }}$ 10^5^	4.6 ${{ \times }}$ 10^5^
	B_2_	0.98245	0.98635	0.97925	4 ${{ \times }}$ 10^4^	4 ${{ \times }}$ 10^4^	4.1 ${{ \times }}$ 10^4^
r_1_, r_2_ ${{ \pm }}$ 5%	A_1_	0.99240	1.0240	0.9623	1.2 ${{ \times }}$ 10^4^	1 ${{ \times }}$ 10^4^	${1}{{.1 \times }}$ 10^4^
	B_1_	1.00258	1.0459	0.9585	4.7 ${{ \times }}$ 10^5^	4.5 ${{ \times }}$ 10^5^	4.8 ${{ \times }}$ 10^5^
	A_2_	0.88074	0.92774	0.83674	5 ${{ \times }}$ 10^5^	${4}{{.2 \times }}$ 10^5^	5.3 ${{ \times }}$ 10^5^
	B_2_	0.98245	1.02445	0.94095	4 ${{ \times }}$ 10^4^	${3}{{.7 \times }}$ 10^4^	${4}{{.2 \times }}$ 10^4^

## Conclusion

In summary, we use DFT calculations with the CASTEP module to obtain electrical, mechanical, and optical properties, as well as the moisture and thermodynamic stability of 2D perovskite PEA_2_PbX_4_ (X = I, Br, and Cl). By comparing the results among PEA_2_PbI_4_, PEA_2_PbBr_4_, and PEA_2_PbCl_4_, we find that PEA_2_PbI_4_ has a higher refractive index in the THz range. In addition, DFT calculations show that all three materials are ductile and flexible. The formation energy calculations indicate that PEA_2_PbI_4_ is thermodynamically more stable due to its higher negative value. Moreover, the surface adsorption energy of water on perovskite reveals that PEA_2_PbI_4_ exhibits higher hydrophobicity, making it more moisture stable than the other two materials. We propose and analyze the excitation of sp-BICs in a novel PEA_2_PbI_4_-based metasurface, where multiple Fano q-BICs with ultra-high Q-factors can be excited. We employ group theory to justify the excitation of BICs and their polarization dependence. Through eigenfrequency and frequency domain simulations, we validate the group theoretical analysis and investigate the near-field distribution as well as the far-field scattering of q-BICs. Based on the results, we can conclude that MTD-EIT-BIC and MQ-BIC can be excited by x-polarized, and ETD-BIC and EQ-BIC by y-polarized incident waves. The effects of meta-atom losses, substrate losses, the finite size of the array, and fabrication tolerance are also investigated. The proposed metasurface with multiple Fano resonances and high efficiency is promising for different photonic applications such as polarization-dependent filters, switches, and wearable sensors.

### Supplementary Information

Supplementary Information.

## Data Availability

The datasets used and/or analyzed during the current study are available from the corresponding author upon reasonable request.
