# Supplementary material for: Design and analysis of a flexible Ruddlesden–Popper 2D perovskite metastructure based on symmetry-protected THz-bound states in the continuum
Source: Sci Rep. 2023 Dec 16;13:22411. doi: 10.1038/s41598-023-49224-9 (PMC10725462; doi:10.1038/s41598-023-49224-9)
Supplement: Supplementary file 1 — Supplementary Information. [file 41598_2023_49224_MOESM1_ESM.docx]

**Supplementary Material**

**Design and analysis of a flexible Ruddlesden-Popper 2D perovskite metastructure based on symmetry-protected THz-bound states in the continuum**

Seyedeh Bita Saadatmand,^1^ Samad Shokouhi,^1^ Vahid Ahmadi,^1,*^ and Seyedeh Mehri Hamidi^2^

^1^Faculty of Electrical and Computer Engineering, Tarbiat Modares University, Tehran, Iran.

^2^Magneto-plasmonic Lab, Laser and Plasma Research Institute, Shahid Beheshti University, Tehran, Iran.

*Corresponding author: [v_ahmadi@modares.ac.ir](mailto:v_ahmadi@modares.ac.ir)

**S1. Details of DFT computational methods**

The DFT calculations to investigate the various properties of two-dimensional perovskite PEA_2_PbX_4_ are performed with the Kohn-Sham equation in the Cambridge Sequential Total Energy Package (CASTEP) module of Materials Studio 2020 software [1]. The pseudopotential describes the electron-ion interaction. Norm-conserving pseudopotential is used to calculate optical properties, and ultrasoft pseudopotential is used for geometry optimization, thermodynamic stability, adsorption energy, and mechanical properties. The Perdew-Burke-Ernzerhof (PBE) exchange-correlation function and generalized gradient approximation (GGA) are used for DFT calculation, which is a semi-local function [2]. The DFT-D correction of Tkatchenko-Scheffler (TS) is utilized to consider the van der Walls interaction between organic and inorganic layers [3]. A 500 eV plane-wave cutoff energy and k-point schemes 3×3×2 (for PEA_2_PbBr_4_ and PEA_2_PbCl_4_) and 4×4×2 (for PEA_2_PbI_4_) are used for meshing the grid and sampling the Brillouin zone in PEA_2_PbX_4_. The low-memory Broyden–Fletcher–Goldfarb–Shanno (LBFGS) minimization technique is performed to optimize crystal structures [4]. Furthermore, finite strain theory computes elastic constants [5]. Convergence thresholds of 4×10^−6^ eV/atom for the total energy, 0.003GPa for the maximum stress, 0.01 eV/Å for the maximum force, and 4×10^−4^ Å for the maximum displacement are adjusted to optimize the perovskite structure geometry.

Table S1. Crystal data for PEA_2_PbX_4_ (X = I, Br, and Cl) [6, 7]

| 2D perovskite | Crystal structure (Space group) | Lattice parameters |
| --- | --- | --- |
| PEA_2_PbI_4_ | Triclinic ($\bar{p_{1}}$) | $\text{a=8.73}\text{6}\text{Å , b=8.737Å, c=16.655Å}$  $\text{α=}{95.20}^{\text{o}}\text{, β=}{99.790}^{\text{o}}\text{,γ=}{90.340}^{\text{o}}$ |
| PEA_2_PbBr_4_ | Triclinic ($\bar{p_{1}}$) | $\text{a=11.61Å , b=11.62Å, c=17.57Å}$  $\text{α=}\text{99.54}^{\text{o}}\text{, β=}\text{105.72}^{\text{o}}\text{,γ=}\text{89.97}^{\text{o}}$ |
| PEA_2_PbCl_4_ | Triclinic ($\bar{p_{1}}$) | $\text{a=11.14Å , b=11.21Å, c=17.69Å}$  $\text{α=}{99.17}^{\text{o}}\text{, β=}\text{104.63}^{\text{o}}\text{,γ=}{89.99}^{\text{o}}$ |


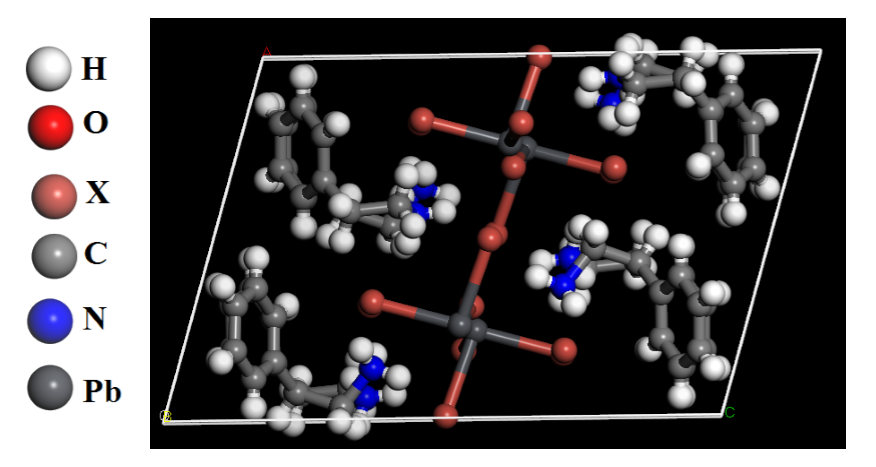


Fig. S1. Schematic of (X = I, Br, and Cl) in DFT analysis

**S2. Details of the mechanical properties**

The mechanical stability of the PEA_2_PbX_4_ (X = I, Br, and Cl) compounds are investigated using the criteria for triclinic crystals [8]. Analysis of the elastic constants presented in Table S2 indicates that all three materials exhibit mechanical stability.

$\text{C}_{\text{11}}\text{>0, }\text{C}_{\text{22}}\text{>0}\text{, }\text{C}_{\text{33}}\text{>0, }\text{C}_{\text{44}}\text{>0, }\text{C}_{\text{55}}\text{>0, }\text{C}_{\text{66}}\text{>0}$ (S1)

$C_{11}+C_{22}+C_{33}+2C_{12}+2C_{13}+2C_{23}>0$ (S2)

$C_{33}{*C}_{55}-C_{35}^{2}>0, C_{44}{*C}_{66}-C_{46}^{2}>0$ (S3)

Table S2. Elastic constants of PEA_2_PbX_4_ (X = I, Br, and Cl)

| 2D perovskite | Elastic constants |
| --- | --- |
| PEA_2_PbI_4_ | C_11_=20.874, C_22_=18.846, C_33_=19.440, C_44_=6.982, C_55_=8.712, C_66_=8.013, C_46_= -2.047, C_35_=-2.244, C_23_=9.307, C_12_=10.741, C_13_=4.483 |
| PEA_2_PbBr_4_ | C_11_=22.856, C_22_=21.887, C_33_=19.668, C_44_=6.999, C_55_=8.663, C_66_=7.861, C_46_=0.020, C_35_=0.523, C_23_=9.392, C_12_=11.192, C_13_=8.523 |
| PEA_2_PbCl_4_ | C_11_=23.809, C_22_=23.087, C_33_=20.487, C_44_=7.007, C_55_=8.561, C_66_=7.625, C_46_=0.558, C_35_=1.373, C_23_=9.473, C_12_=11.222, C_13_=9.391 |

**S3. Details of water adsorption energy**

The structures required to calculate the adsorption of water (H_2_O) molecules on the surface of PEA_2_PbX_4_ for DFT calculations can be seen in Fig. S2. In the calculations, we consider the case where the oxygen atom of H_2_O is bonded to the hydrogen atom of the aromatic ring of PEA in PEA_2_PbX_4_.


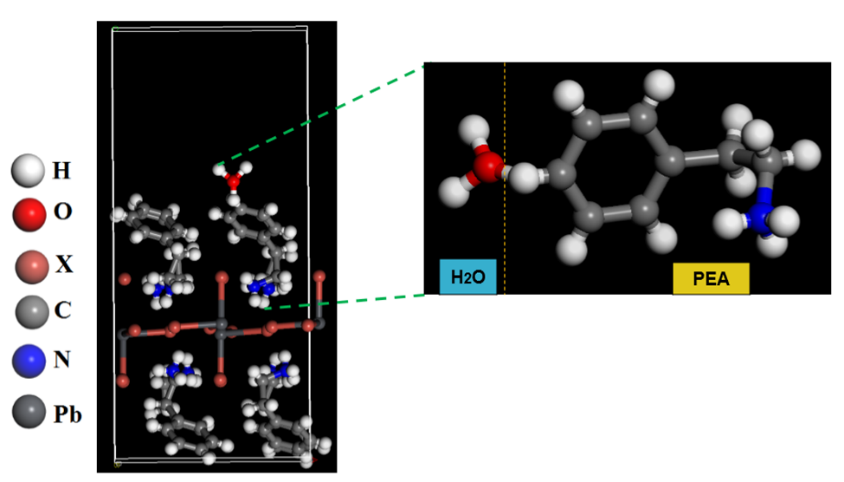


Fig. S2. H_2_O on the surface of PEA_2_PbX_4_

**S4. Symmetry analysis**

One way to reduce the C_4v_ group to its lower subgroup, C_2v_, is by changing the inner circular ring of the UC to an ellipse. However, according to Tables S3 and S4, this reduction results in IRREP A_1_ (B_1_) of C_4v_ being reduced to A_1_ (A_1_) of group C_2v_, and IRREP A_2_ (B_2_) of C_4v_ being reduced to A_2_ (A_2_) of group C_2v_. This also means that the orthogonal nature of the previously mentioned dark mode fields with respect to the electric field of the incoming wave remains unchanged, rendering their excitation unachievable even with this decrease (Fig. S3). As a result, the four symmetry-protected BIC dark modes remain unchanged.

Table S3. IRREPs of group C_2v_

| C_2v_ | e | C_2_ | σ_x_ | σ_y_ | Mode order |
| --- | --- | --- | --- | --- | --- |
| A_1_ | 1 | 1 | 1 | 1 | dark |
| A_2_ | 1 | 1 | -1 | -1 | dark |
| B_1_ | 1 | -1 | 1 | -1 | bright |
| B_2_ | 1 | -1 | -1 | 1 | bright |

Table S4. Symmetry degeneration of C_4v_ [9]

| C_4v_ | C_2v_ | C_s_ |
| --- | --- | --- |
| A_1_ | A_1_ | A |
| A_2_ | A_2_ | B |
| B_1_ | A_1_ | A |
| B_2_ | A_2_ | B |
| E | B_1,_ B_2_ | A, B |

Fig. S3. Electric field profiles of C_2v_ structure

The proposed asymmetry structure (C_s_) perturbs the modes, as shown in Fig. S4. The arrow sizes are slightly varied to indicate the lack of symmetry with respect to that axis. It can be observed that breaking the symmetry along the y-axis results in a corresponding break in the symmetry of modes along that axis, while the symmetry along the x-axis remains intact. As shown in the figure below, after disturbance, modes A_1_ and A_2_ are coupled to x and y polarizations, respectively, which shows the possibility of their selective excitation. These results also apply to modes B_1_ and B_2_.

Fig. S4. Illustration of the coupling between plane waves and perturbed modes A_1_ and A_2_. Electric fields are represented by arrows.

**S5. Band structures of PEA_2_PbX_4_**

In Fig. S5, the band structure of PEA_2_PbX_4_ (X = I, Br, and Cl) is shown by DFT analysis. The band gaps (E_g_) are 2.130 ev, 2.584 ev, and 3.148 ev for PEA_2_PbI_4_, PEA_2_PbBr_4_, and PEA_2_PbCl_4_, respectively. It is evident that E_g_ increases when changing the halide from I to Br and then to Cl.

Fig. S5. Band structure and E_g_ of (a) PEA_2_PbI_4_, (b) PEA_2_PbBr_4_, and (c) PEA_2_PbCl_4_

**S6. Mode analysis**

Fig. S6. Electric and magnetic field patterns for bright modes under (a) y-polarization, and (b) x-polarization at d=0.

Fig. S7. (a) Displacement current distribution of mode A_1_, and (b) Magnetic field distribution of mode A_2_. Gray ribbons show displacement currents and black ribbons show magnetic fields.

**S7. Multipole decomposition**

The most important moments are defined by [10, 11]:

Electric dipole (ED)

 (S4)

Magnetic dipole (MD)

 (S5)

Magnetic toroidal dipole (MTD)

 (S6)

Electric toroidal dipole (ETD)

 (S7)

Electric quadrupole (EQ)

 (S8)

Magnetic quadrupole (MQ)

 (S9)

In these equations, *j* is the displacement current density, *ω* represents the angular frequency, and *c* and *r* represent the light speed and position vectors, respectively. The Dirac delta function is represented by *δ* and the subscripts *α* and *β* can take on the values x, y, or z. The calculation of the scattering power for each multipole moment is achievable by referencing [12, 13].

**S8. Losses effect on reflectance curves**

Fig. S8. Evolution of the reflectance curves versus metaatom’s extinction coefficient for (a) x-polarized incident wave, (b) y-polarized incident wave at d= 0.5 µm

**References**

1. Kohn, W. and L.J. Sham, *Self-consistent equations including exchange and correlation effects.* Physical Review, 1965. **140**(4A): p. A1133.

2. Perdew, J.P., K. Burke, and M. Ernzerhof, *Generalized gradient approximation made simple.* Physical Review Letters, 1996. **77**(18): p. 3865.

3. Tkatchenko, A. and M. Scheffler, *Accurate molecular van der Waals interactions from ground-state electron density and free-atom reference data.* Physical Review Letters, 2009. **102**(7): p. 073005.

4. Pfrommer, B.G., et al., *Relaxation of crystals with the quasi-Newton method.* Journal of Computational Physics, 1997. **131**(1): p. 233-240.

5. Giustino, F., *Materials modelling using density functional theory: properties and predictions*. 2014: Oxford University Press.

6. Du, K.-z., et al., *Two-dimensional lead (II) halide-based hybrid perovskites templated by acene alkylamines: crystal structures, optical properties, and piezoelectricity.* Inorganic Chemistry, 2017. **56**(15): p. 9291-9302.

7. Menahem, M., et al., *Strongly anharmonic octahedral tilting in two-dimensional hybrid halide perovskites.* ACS Nano, 2021. **15**(6): p. 10153-10162.

8. Wu, Z.-j., et al., *Crystal structures and elastic properties of superhard Ir N 2 and Ir N 3 from first principles.* Physical Review B, 2007. **76**(5): p. 054115.

9. Overvig, A.C., et al., *Selection rules for quasibound states in the continuum.* Physical Review B, 2020. **102**(3): p. 035434.

10. Liu, X., et al., *Dual-toroidal dipole excitation on permittivity-asymmetric dielectric metasurfaces.* Optics Letters, 2020. **45**(10): p. 2826-2829.

11. Wang, W., J. Qi, and B. Li, *Double toroidal switches based on the different multipole responses in the all-dielectric metasurface.* Journal of Nanophotonics, 2020. **14**(3): p. 036010-036010.

12. Alaee, R., C. Rockstuhl, and I. Fernandez-Corbaton, *An electromagnetic multipole expansion beyond the long-wavelength approximation.* Optics Communications, 2018. **407**: p. 17-21.

13. Alaee, R., C. Rockstuhl, and I. Fernandez‐Corbaton, *Exact multipolar decompositions with applications in nanophotonics.* Advanced Optical Materials, 2019. **7**(1): p. 1800783.
